# Notes and Comments

**Published:** 1901-02

**Authors:** 


### Notes and Comments.[1]

Are the Colleges Responsible for the Quacks?—We recall Dr. Barrett expressing himself on this subject as follows: "It is not too much to say that our professional reputation must be what our colleges make it. We are the educators of those who are to be the leaders in the future. The next generation of dentists will be what we shall make it. Legislatures may pass laws to regulate and restrict dental practice, but the stream can rise no higher than the fountain-head, and the practitioner of to-morrow must get his training and derive his professional knowledge from the schools of to-day. The colleges are the fountain-head, and the stream will be limpid or foul according to whether we purify or contaminate it."

This is true in a measure, but not in the breadth of the asser-

[1] The assistant editor solicits contributions for this department,—new methods, new remedies and formulas, or any short practical note which may prove of value to the practitioner or student. Address 1338 Walnut Street, Philadelphia.

tion. The colleges, of course, should have the right moral stamina in them, or they should not exist. And teachers should endeavor to be an inspiration to their students, but it is too much to say that the college is responsible for the conduct of men after they leave her doors. If out of a class of one hundred students, five or even ten should go wrong in practice, and if Dr. Barrett is right, should the same school, the same teachers, the same influences, produce the ninety good men? This calls to mind an experience which occurred some years ago. After speaking to a class of students upon ethical matters, several members were expressing their views, when one man remarked that, notwithstanding all that had been said by his teachers, he and his chum intended to conduct an advertising establishment after they graduated, as they "intended to practise dentistry to make money." These men passed excellent examinations, and therefore graduated. They are to-day conducting an advertising practice. *Is their alma mater responsible for it?* It is not from a lack of teaching or example,—these are evident in every reputable college,—but in spite of them, that many of our men go wrong. The charlatan, we believe, is as surely a self-made man as many a reputable practitioner. Every one has it within himself to choose whether he is to rank with the blatant quack or whether he is to be respected. In the legal profession they have a way of keeping the would-be quack in line, by disbarring a man for unprofessional conduct. The bar associations are ever active in their efforts to purge their profession of dishonest and unworthy members. We suffer from the lack of some such means to turn out and shut out men who degrade an honorable calling.

---

INSTRUCTION OF MEDICAL STUDENTS IN THE PRINCIPLES OF DENTISTRY.—In a paper read before the American Medical Association, and published in the *Dental Cosmos,* Dr. M. L. Rhein says that the question narrows itself down to the advisability of the medical undergraduate being acquainted with principles of dentistry as they bear on general medicine. The general practitioner should appreciate fully the process of dentition in its relation both to local and constitutional results. Equally as important is it that he should be able to distinguish an incipient alveolar abscess from tic douloureux, simple caries from caries complicated by exposure of the pulp, or the inflammation attending the eruption of a third molar from that caused by follicular tonsillitis. He should be taught to know that more teeth are lost through disease of the peridental membrane than through all other pathological conditions of the mouth combined, and that prophylactic measures tending to preserve this membrane are of vital importance. He should be made cognizant of the intimate relationship existing between the general nutrition and proper mastication, so as to realize when artificial teeth are required, and, if supplied, whether they are properly inserted. Such knowledge implies a proper understanding of the normal occlusion of the upper and lower teeth; it also leads to the appreciation of the value of orthodontia as a corrective for malocclusion.

A proper appreciation of the foregoing facts necessitates that medical undergraduates be taught dental embryology, anatomy, histology, and pathology, in order that these principles should form a foundation for a correct clinical observation of oral conditions. This will enable the general practitioner to serve best both his own interests and the interests of his patients, and at the same time tend to elevate the standing of the dental specialty.

